# Case Report of Placenta Accreta Spectrum and Arteriovenous Malformations with Successful Preservation of Fertility After Birth

**DOI:** 10.3390/diagnostics14222538

**Published:** 2024-11-13

**Authors:** Constantin-Cristian Vaduva, Laurentiu Dira, Sidonia Maria Sandulescu, Cristian Constantin, Elena Silvia Bernad, Dana Maria Albulescu, Mircea-Sebastian Serbanescu, Lidia Boldeanu

**Affiliations:** 1 Department of Obstetrics and Gynecology, Filantropia Clinical Hospital, University of Medicine and Pharmacy, 200143 Craiova, Romania; cristian.vaduva@umfcv.ro (C.-C.V.); laurentiu.dira@yahoo.com (L.D.); ssidoniam@yahoo.com (S.M.S.); 2 Department of Obstetrics, Gynecology and IVF, HitMed Medical Center, 200130 Craiova, Romania; 3 Department of Radiology, County Clinical Emergency Hospital, University of Medicine and Pharmacy, 200642 Craiova, Romania; cristian.constantin2364@gmail.com; 4 Department of Obstetrics and Gynecology, Victor Babeș University of Medicine and Pharmacy, 300041 Timisoara, Romania; 5 Clinic of Obstetrics and Gynecology, “Pius Brinzeu” County Clinical Emergency Hospital, 300723 Timisoara, Romania; 6 Center for Laparoscopy, Laparoscopic Surgery and In Vitro Fertilization, Victor Babeș University of Medicine and Pharmacy, 300041 Timisoara, Romania; 7 Department of Anatomy, University of Medicine and Pharmacy, 200349 Craiova, Romania; dana.albulescu@umfcv.ro; 8 Department of Pathology, Filantropia Clinical Hospital, University of Medicine and Pharmacy of Craiova, 200143 Craiova, Romania; mircea_serbanescu@yahoo.com; 9 Department of Microbiology, County Clinical Emergency Hospital, University of Medicine and Pharmacy, 200642 Craiova, Romania; lidia.boldeanu@umfcv.ro

**Keywords:** uterine arteriovenous malformation (UAVM), placenta accreta spectrum (PAS), color Doppler ultrasound (CDUS), uterine artery embolization (UAE), postpartum hemorrhage (PPH), case report

## Abstract

Uterine arteriovenous malformations (UAVMs) that occur after birth are a rare cause of late postpartum hemorrhage. Acquired UAVMs usually occur in conjunction with pathology of the placenta. In the spectrum of placenta accreta (PAS), subinvolution of the placental bed plays an important role in its pathophysiology. We present a case of UAVM in a pregnant woman with PAS who presented with marked metrorrhagia after delivery, which was treated with classical management. Then, 35 days later, she presented to the emergency room with severe metrorrhagia. As it was suspected that she had placental remnants, an instrumental uterine control was performed, but the bleeding persisted, requiring further uterine packing and blood administration. Later, uterine artery embolization was performed with good results. Color Doppler ultrasound, magnetic resonance imaging, and angiography were the methods with the greatest diagnostic value. The differential diagnosis was as complex as the treatment. We hypothesize that UAVM may develop from minimal residual PAS in this late postpartum period. Moreover, they may recover rapidly after local surgical ablation. Considering the clinical condition, hemodynamic status, and desire to preserve fertility, we were able to avoid a hysterectomy, which is often chosen in such cases of severe, life-threatening bleeding complications.

## 1. Introduction

Placenta accreta spectrum (PAS) refers to abnormal trophoblast invasion of the uterine wall. PAS is associated with a higher risk of severe bleeding during pregnancy.

In 50% of cases, PAS remains undetected until birth [1]. The time of birth and the immediate postpartum period are the most affected [2]. As the placenta cannot detach spontaneously, massive bleeding can occur, often requiring an emergency hysterectomy [3,4]. After detachment of the placenta, postpartum hemorrhage (PPH) is usually caused by retained products of conception (RPOC). Uterine vascular malformations (UAVMs) are also considered a rare cause. Although the prevalence of uterine UAVM is not known, a prospective analysis of 959 consecutive patients who had either an abortion or delivery found that the incidence of true UAVM was 0.1%, with a significantly higher rate following abortion [5]. Primary PPH is defined as blood loss of more than 500 milliliters within the first 24 h after delivery, while secondary PPH is defined as excessive uterine bleeding that occurs between 24 h and 6 weeks after delivery. UAVM is responsible for secondary PPH [6]. UAVMs are pathologic anastomoses that develop between the uterine arterioles and the uterine venous plexus without intervening capillaries. Cases of UAVM associated with PAS can lead to severe PPH complications [7]. Due to the severe bleeding and the peculiarities of uterine hemostasis, the therapeutic approach becomes a challenge for the medical team.

We present the case of a parturient with PAS associated with the development of UAVM. The birth proceeded naturally, but significant PPH accompanied the immediate and late delivery. However, fertility was preserved by uterine artery embolization (UAE).

## 2. Case Presentation

A 36-week pregnant woman admitted to the hospital via emergency service delivered a live fetus weighing 2700 g and Apgar 9 at 37 weeks’ gestation nearby. Initially, we had no data on her medical history because she was treated at another medical facility. During the ultrasound examinations (US) in the 32nd week of pregnancy, a placenta previa and a bilobate were detected by another medical team.

The pregnant woman had experienced a eutopic birth four years previously.

As the placenta was not delivered (retained placenta), we performed a manual extraction of the placenta. Inspection of the placenta revealed tissue deficits, so we performed a postpartum instrumental check with suspected placental retention. No tears of the cervix or vagina were detected during the local inspection. PPH was severe, with signs of hemorrhagic shock. Hemoglobin (Hb) decreased from 10.8 g/dL to 9.2 g/dL and hematocrit (Ht) from 31.4% to 27% half an hour postpartum. The amount of blood lost was estimated to be approximately 1945 mL using the formula for allowable blood loss (ABL) [8]. We administered a hydro electrolyte replacement and uterotonic drugs. Finally, we started uterine gauze packing. The patient was transferred to the intensive care unit, where she received two units of red blood cell concentrate and one unit of plasma. The next day, she received another unit of red blood cell concentrate, and the Hb fell to 7 g/dL and the Ht to 20.2%. The next day, the uterine gauze packing was removed as the metrorrhagia ceased and the patient was hemodynamically balanced. After treatment of acute anemia, the postpartum period was favorable. On the US examination, we found uterine hematometry. After 6 days, Hb = 9.4 g/dL and Ht = 27.6%. The patient was discharged to the outpatient clinic with physiological lactation and anemia-inhibiting treatment.

The patient was called in for regular check-ups in the late postpartum phase but presented for a single follow-up examination after one week. At this outpatient check-up, the doctor was unable to detect any pathological changes in the uterus on ultrasound.

**Figure 1:**
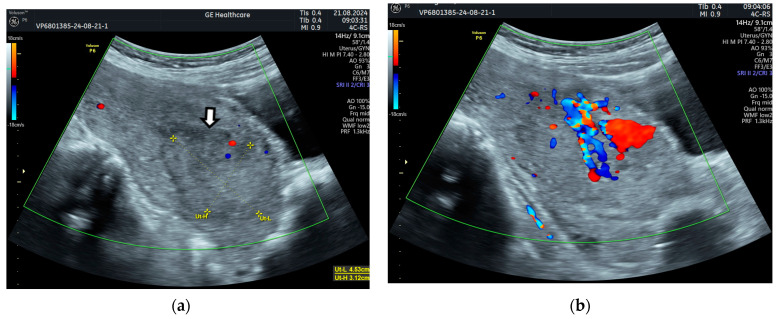
Vaginal ultrasound image of the uterus: (**a**) arrows show the lower part of the uterine cavity filled with a homogeneous content (hematometry), with a hypoechoic linear zone at the upper edge adjacent to the myometrium; (**b**) vascular formations in the uterine cavity on color Doppler.

Then, 35 days after delivery, she presented to the emergency room with heavy bleeding. The US examination showed a subinvoluted uterus with trilaminar endometrium in the upper half of the uterine cavity but with a relatively homogeneous formation of about 5/4 cm occupying the lower part of the uterine cavity; the US image resembled the contents of an endometriotic ovarian cyst [9]. Color Doppler ultrasound (CDUS) showed vascular formations in the uterine cavity (Figure 1).

**Figure 2:**
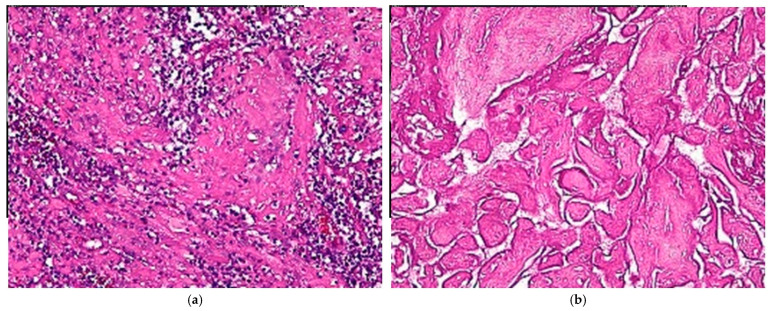
The histological images of the uterine biopsy show (**a**) decidua and fibroconjunctival fragments with structured and inflammatory necroses; (**b**) sclerohyalinized placental villi with structured necroses (HE × 20).

The initial beta-HCG value was 16 mIU/mL. As RPOC was suspected and PPH was important, we urgently performed uterine aspiration and instrumental examination of the uterine cavity, which confirmed the presence of a cavity filled with clotted blood (hematometry). A hysteroscopy was not performed because the clot in the uterus was large and the bleeding was severe, requiring emergency surgery. Systematic scraping of the walls of this cavity revealed no placental debris but only small fragments of endometrium. These fragments were sent to pathology, stained with hematoxylin–eosin (HE) and examined under a light microscope, as described in a previous article [10]. They were examined by a pathologist who specializes in endometrial pathology. Unfortunately, this result was not received from the histology laboratory until 10 days later. The subsequent pathological examination revealed necrobiotic decidua and fragments of placental villi of different sizes, which were sclerohyalinized and necrobiotic (Figure 2).

After the curettage, the uterine bleeding became even heavier. As the bleeding continued to be heavy, uterotonic drugs (methylergonovine maleate and carbetocinum) were administered and a uterine gauze dressing was applied. The Hb value fell from 9.5 g/dL to 7.4 g/dL and the Ht value from 27.3% to 21.6%. The estimated blood loss was 2500 mL. On the day after curettage, the beta-HCG dropped to 9 mIU/mL.

As the metrorrhagia stopped, the gauze dressing was removed on the second day. In the next few days after curettage, 4 units of red blood cell concentrate and 1 unit of blood plasma were administered.

**Figure 3:**
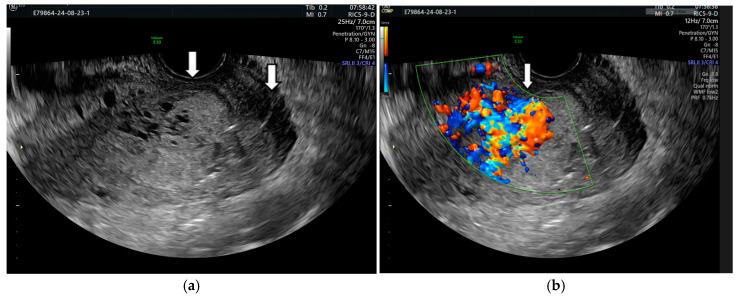
Vaginal ultrasound image of the uterus. Arrows show (**a**) multiple anechoic areas in the myometrium and uterine cavity with hematometry; (**b**) marked vascularity with a colored mosaic pattern.

Three days after curettage, the beta-HCG level decreased to 4 mIU/mL, and the myometrium showed a heterogeneous appearance with numerous anechoic areas. These anechoic formations could also be observed inside the uterine cavity. The CDUS showed an intense signal with numerous tortuous arterial and venous vessels with a serpentine course and varying flow rates. The appearance resembled a vascular sphere (Figure 3).

**Figure 4:**
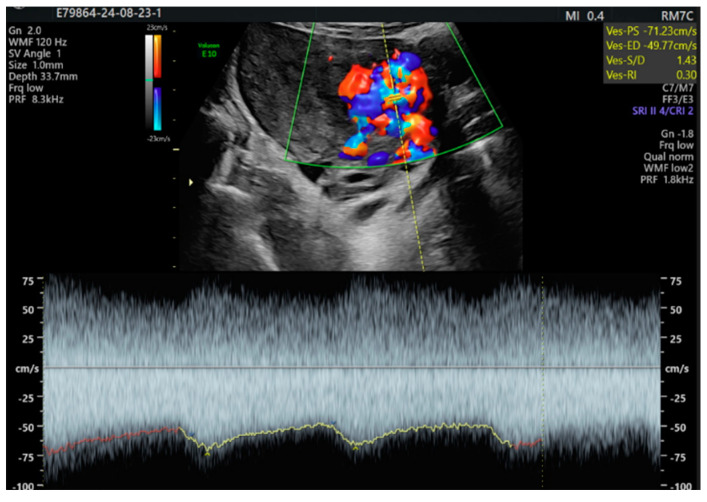
Pulsatile Doppler US image with turbulent flow: the spectral Doppler shows a flow with low resistance in the arterial vessel.

The pulsatile Doppler US showed that the arterial vessels had a high peak systolic velocity (PSV = 71 cm/s) and a low resistance index (RI = 0.30) (Figure 4).

**Figure 5:**
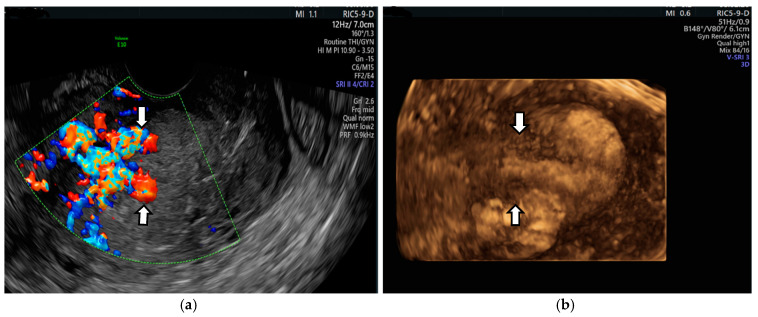
Vaginal ultrasound image of the uterus 3 days after curettage. Arrows show (**a**) vascular pedicles in the uterine cavity—color Doppler; (**b**) the same image in three-dimensional ultrasound.

The color and 3D US images showed vascular pedicles that protruded into the uterine cavity (Figure 5).

The presence of these vascular pedicles inside the uterine cavity after an insistent curettage of the walls of the uterine cavity surprised us.

**Figure 6:**
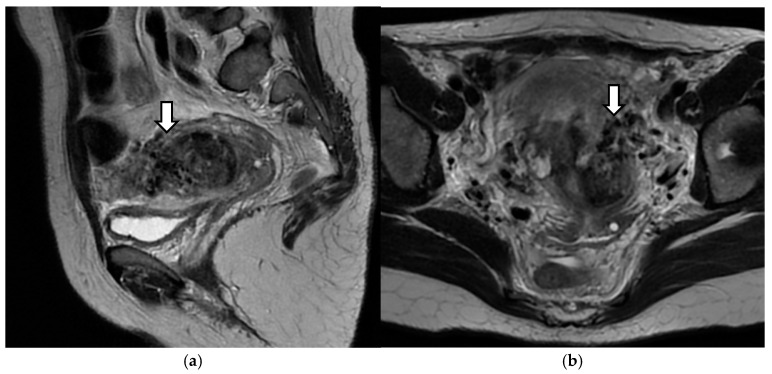
Dynamic magnetic resonance images of the pelvis. Arrows show numerous signal voids in the uterine wall: (**a**) longitudinal section; (**b**) transverse section.

Dynamic magnetic resonance imaging (MRI) showed a heterogeneous lesion of 4/6/5 cm in which the distinction between endometrium and myometrium was abolished and the serous lesion was preserved. This formation occupied the lower 2/3 of the uterine cavity and protruded at the level of the proximal cervix. Numerous peri- and intralesional serpiginous hyposignaling structures were described that appeared to drain into the bilateral parametrial venous plexuses (Figure 6).

The MRI images raised the suspicion of choriocarcinoma.

**Figure 7:**
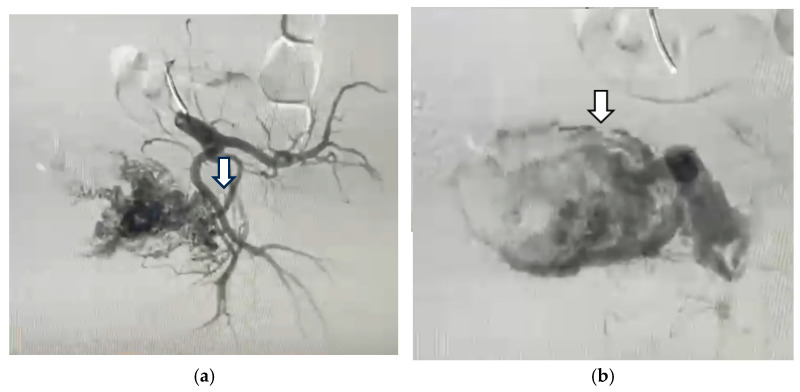
Arteriography of the left internal artery shows (**a**) left uterine artery; (**b**) hypervascular mass next to and around the uterine cavity.

**Figure 8:**
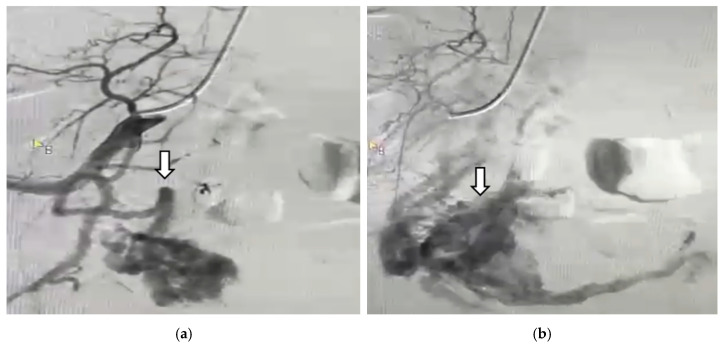
Arteriography of the right internal artery shows (**a**) right dilated uterine artery; (**b**) hypervascular mass next to and around the uterine cavity.

The patient was referred for angiography. Selective bilateral angiography of the common iliac arteries revealed a UAVM consistent with the flow gaps seen on the MRI. The angiographic appearance included supply vessels from bilateral uterine arteries, as seen in hypervascular masses, with early drainage of the UAVM into the iliac veins (Figure 7 and Figure 8).

**Figure 9:**
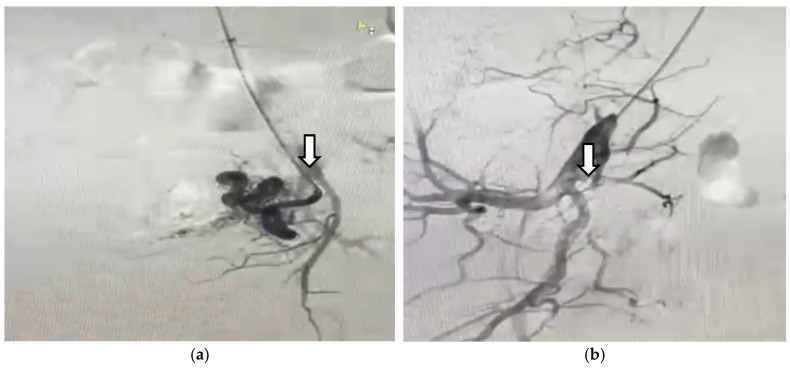
Arteriography after selective uterine artery embolization shows no hypervascular mass next to the (**a**) left uterine arteries; (**b**) right uterine arteries.

The hypervascular masses disappeared bilaterally after selective UAE (Figure 9).

Finally, the decreased beta-HCG level after curettage and the late histopathologic results raised the suspicion of RPOC. However, the vascular US Doppler and MRI as well as the angiographic aspect were mainly specific for UAVM.

**Figure 10:**
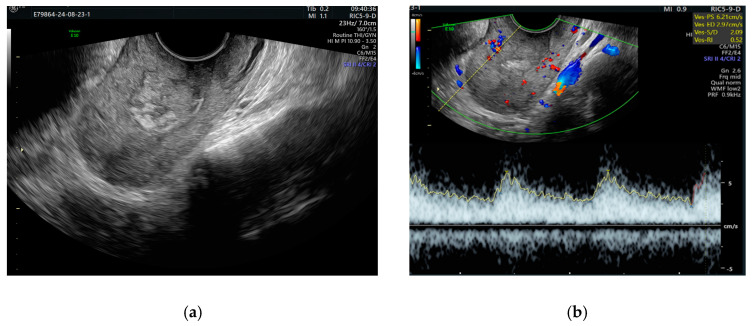
Vaginal ultrasound 4 weeks after embolization shows (**a**) intrauterine heterogeneous contents; (**b**) normal vascular indices.

After 5 days at discharge, the patient had a Hb of 10.7 g/dL, an HT of 30.6%, and no signs of metrorrhagia. The patient, concerned about her illness, refused to breastfeed. We recommended hypoestrogenic treatment with Relugolix derivative (Ryeqo, Gedeon Richter Inc., Budapest, Hungary) 40 mg, 1 dose per day. At the following consultations, which were performed at 2-week intervals, the patient developed favorably without metrorrhagia occurring. Ultrasound examination performed at 4 weeks showed a linear endometrium and the presence of a 2.5 cm formation with heterogeneous contents, without any Doppler US signal, probably with organized clots and fibrosing vascular lesions. The surrounding uterine wall showed vascularization with normal distribution and vascular index (PSV = 11.47 and RI = 0.52) (Figure 10).

## 3. Discussion

### 3.1. Physiopathology

Placenta accreta was first described in 1937 by a gynecologist and pathologist in the USA [11]. Depending on the degree of invasion of the placental tissue, several types are distinguished: placenta accreta; increta; and percreta, when the trophoblast invades the basement membrane, the uterine wall, or the serum and possibly also the neighboring organs [4,12]. The more comprehensive term placenta accreta spectrum (PAS) includes all three morphological entities. The chorionic villi adhere to or penetrate deeply into the myometrium; the basal decidua is not present in this area [1,13,14]. Due to the increasing number of cesarean sections, PAS has recently become more common, with an incidence of 1 in 500 births [12,15].

In our case, the pathological aspect described in Figure 5, the low beta-HCG level, the clinical context, and the history of the previa and bilobate placenta led us to retrospectively diagnose PAS. An additional analysis of the placenta previa and bilobate could have helped us, but the patient came to the emergency room and subsequently delivered. Originally, we had no data on her medical history, as she was cared for in another medical establishment.

PAS may be associated with uterine arteriovenous anastomoses (UAVMs). The first series of documented cases of UAVM with PAS was described in 2011 [16]. Subsequently, more cases were reported, but the vast majority of UAVMs occurred after abortion [17]. Cases of UAVM occurring after childbirth are rare [18]. UAVMs are pathologic anastomoses between the arterioles and the myometrial venous network.

The explanation for the occurrence of UAVM after birth would be the subinvolution of the placental bed, which causes a lack of sclerosis of the vessels at this level. The cause of subinvolution is not known, but it is hypothesized that there is an abnormal immunological relationship between the fetal trophoblasts and the maternal uterine tissue [19]. Syncytiotrofoblast and intermediate trophoblast, which can erode and infiltrate to establish blood flow, may play essential roles in its pathophysiology. This suggests that both UAVM and PSA may be involved in the pathologic process of invasive placental disease [16,20]. The authors believe that the development of UAVM should be considered as a component of aberrant placental invasion and not as an independent entity if trophoblastic activity has previously occurred in utero [21,22]. This aspect also applies to our UAVM case after PAS, in which histopathologic examination revealed the presence of tiny RPOC.

### 3.2. UAVM Diagnosis

Menorrhagia or metrorrhagia may occur gradually or unexpectedly. Bleeding is often heavy and sporadic. It is a secondary, late PPH [6]. Originally, UAVM was diagnosed histologically based on uterine samples taken after a hysterectomy.

Nowadays, diagnoses are made using imaging techniques. US is the most important diagnostic method used [23]. UAVM presents multiple hypo/anechogenic cystic or tubular lesions that occur in the structure of the myometrium. These formations do not show post-injury attenuation as in adenomyosis [24,25]. The endometrium is not affected by these formations. CDUS shows a highly vascularized lesion consisting of tortuous vessels and having a tortuous appearance with a turbulent, multidirectional outflow [26]. They often resemble a “vascular ball” in appearance. Low resistance index (RI) values and a pulsatility index with a high peak systolic flow velocity (PSV) are characteristics [27]. The systolic and diastolic velocities are four to six times higher in UAVM: the PSV is between 25 and 110 cm/s, with a mean of 60 cm/s, and a resistance index of 0.27–0.75, with a mean of 0.41 [28].

MRI shows hypervascular arterial-dominated flow in the myometrium with marked enhancement on post-contrast imaging, interruption of the junctional zones, a prominent parametrial vessel, and a large uterus with a focal mass [26].

Three-dimensional CT is used to determine the degree of involvement, to rule out extrauterine involvement, and to localize the feeding vessels or uterine arteries [29]. It is usually reserved for urgent cases when the patient has metrorrhagia and MRI does not provide sufficient resolution, or before surgery. One of the advantages of CT over MRI is the better resolution in areas near to the bone or intestine.

The gold standard for the diagnosis of UAVM is CT angiography, which reduces the radiation dose and examination time and reduces the need for contrast agents in patients in whom embolization is planned. As in our case, it shows early venous return into a dilated vein and a dilated, tortuous uterine artery supplying a hypervascular uterine mass. CT angiography is preferred in unstable patients with heavy bleeding or in patients in whom MRI is contraindicated [30]. As this technique is generally associated with high radiation exposure, digital subtraction angiography can be performed with less ionizing radiation and contrast medium than conventional examinations and allows for three-dimensional reconstructions of the examined vascular system [31]. Angiography is required for therapeutic embolization to avoid or delay surgical resolution [26,32].

Hysteroscopy is another diagnostic method and is also important for the differential diagnosis of RPOC. It is useful when the UAVMs are located directly beneath the endometrium and appear as conspicuous pulsating, bumpy vascular structures on the surface of the uterine cavity [33].

### 3.3. Differential Diagnosis

In the very early stages of pregnancy, a hypervascular appearance with turbulent flow occurs due to vasodilation in the spiral artery [34].

Disorders of intrauterine vascularization after birth or miscarriage also include malignant tumors originating from the villi (e.g., invasive moles and trophoblastic tumors on the placenta). These can be distinguished by abnormally high concentrations of HCG and placental lactogen hormones [35].

A pseudoaneurysm that arises near the artery is a “pool of blood” in the sense that it lacks a vessel wall structure [34,36]. On CDUS, it appears as an intrauterine hypoechoic mass with a yin–yang sign [37]. In certain situations, pseudoaneurysms have spontaneously resolved [38].

Most cases of enhanced myometrial vascularity are not true UAVMs but RPOCs [36]. In most cases, dilation and curettage result in the whole elimination of this RPOC, cessation of vaginal bleeding, and rapid resolution of the increased myometrial vascularity [37].

They are candidates for elective treatment as they can regress spontaneously within a few months [39,40]. However, neovascularization in RPOC is much less than in UAVM and does not have the characteristics of arteriovenous strains [22]. In RPOC, the differential diagnosis of UAVM is based on an ultrasound examination that shows intracavitary heterogeneous content but also on the histopathological examination of the product extracted by curettage or hysteroscopy. In RPOC, the neovascularization is limited to contact of any remnants with the myometrium and no pulsatile perfusion in the remnant itself [22].

In UAVM, CDUS shows abnormal vascularization confined to the myometrium and rapid blood flow compared to RPOC. The flow is continuous during both the systolic and diastolic cycles with high systolic velocity [20,22]. Beta-HCG is negative, and CT angiography shows the presence of opacified myometrial formation with vascular network and early venous return [33,41].

Since we were able to diagnose the UAVM just 35 days after birth, we suppose that these neovascular lesions may grow from minimal remnants of PAS in this late postpartum period. Given the important limitations and influencing factors, this result should be interpreted with caution; further investigations are needed to elucidate this aspect.

Some authors claim that if the remaining villi necrotize and fibrin forms, the pathological condition known as placental polyps may occur. When the polyp detaches, the patient experiences severe bleeding [42]. Perhaps this was our case, and we unexpectedly observed these intrauterine vascular pedicles recovered 3 days after a systematic and persistent curettage of the uterine cavity. This aspect must be clarified by further studies.

### 3.4. Treatment

Treatment options depend on the clinical condition, the hemodynamic status, the location and size of the lesions, and the desire to preserve fertility.

As in our case, the primary goal was to keep the hemodynamics stable and control the bleeding. Blood transfusions and uterocervical–vaginal tamponade were required [43]. A balloon tamponade can be useful to stop the bleeding and stabilize the patient [6]. We performed this tamponade with intrauterine gauze packing as we did not have a Bakri balloon available in our settlement.

In patients with minimal metrorrhagia, positive results are described when drug treatment is performed. Treatment with danazol (400 mg once daily) for 12 weeks would lead to UAVM thrombosis [44]. Other drug therapies include oral contraceptives for at least three months or methotrexate therapy. The use of gonadotropin-releasing hormone agonists (GnRH-a) or, more recently, antagonists (GnRH-ant) are further modalities of drug treatment [45]. After embolization, we carried out therapy with an oral GnRH-ant with good results. In a recent review, Rosen A. gave a success rate of over 80% for drug treatment: 85% for progesterone derivatives, 89.3% for GnRH-a, and 90% for methotrexate [46]. As in our case, we should not forget the importance of taking uterotonics in emergencies [47].

Since the thin layer of endometrium covering the anomaly can easily be cut, uterine curettage can lead to heavy bleeding. This should therefore be avoided [48]. However, if RPOCs are present, surgical removal can often resolve the coexisting UAVM. This happened in our case when we suspected RPOC.

An UAE can be performed on patients of reproductive age in whom fertility is to be preserved. There are authors who claim that UAEs can be used to preserve fertility if the bleeding caused by UAVMs is reduced [44,49]. We have shown that this wish is also possible in the case of massive bleeding. If the bleeding recurs, an UAE can be performed again [50,51,52]. More common complications after UAEs are pelvic pain and fever [53]. Although rare, pulmonary embolism is a problem in the treatment of UAVMs [54].

A recent review of fertility after a uterine artery embolization (UAE) showed that 77% of pregnancies were uneventful, but abortion was the most common complication, possibly due to ischemic damage to the endometrium. The time between the embolization and pregnancy outcome varied from 2 months to 5 years, with no recurrence of arteriovenous malformations (AVMs) reported during or after pregnancy [55]. The presented case was extremely rare, and since it occurred recently, we did not observe any recurrence of UAVM 3 months after the embolization. UAE has minimal impact on the occurrence of future pregnancies or the development of fertilization products [56,57,58]. Other studies suggest a higher risk of infertility, premature ovarian failure, and uterine synechiae after UAE, but these results have a low level of evidence [59]. However, we believe that premature ovarian failure can currently be overcome by in vitro fertilization with donor eggs.

Surgical treatment is useful when a drug treatment or UAE is contraindicated [45].

To prevent catastrophic bleeding, a hysterectomy should be performed immediately if vaginal blood loss is still significant after the UAE [52]. There is a possible difference in transfusion but no difference in hysterectomy rate between the two strategies: UAE or hysterectomy.

Surgical treatment initially consists of ligation of the iliac artery or uterine artery and hysterectomy [60]. However, ligation of the iliac artery may lead to the formation of collateral anastomoses, rendering the procedure ineffective [61].

UAVMs are morphogenetic defects with stable cellularity, structural organ defects, and no spontaneous regression [22]. Since spontaneous regression is not possible, they must be treated immediately. If the PSV is >83 cm/s, hysterectomy is the saving measure [56]. For many authors, hysterectomy is the treatment of choice for UAVMs after PAS. The reason for this is the difficulties associated with abnormal placentation, RPOC, and UAVM—all of which cause acute and severe bleeding that can endanger the patient’s life [1,17,40,62,63].

Laparoscopic bipolar coagulation of the uterine, ovarian, and mesosalpinges, as well as the bilateral uterine arteries are additional options to hysterectomy. Simultaneous blockage of the bilateral uterine arteries and ovarian ligaments can greatly increase the risk of endometrial atrophy and impair ovarian function, which has a negative impact on fertility [64]. Other surgical techniques include removal or suturing of the UAVM [51,64].

## 4. Conclusions

Postpartum UAVMs are very rare but can be associated with severe bleeding that is difficult to control. They occur more frequently after conservative treatment of PAS. Since the endometrial fibrinoid membrane is absent in PAS, the abnormal location of the trophoblastic tissue may favor angiogenesis and the development of UAVMs.

The acute, sometimes dramatic manifestation of UAVM must be considered at some distance from delivery with PAS. In our case, UAVM was diagnosed 35 days postpartum. It is possible that UAVMs resulting from PAS with RPOC may grow in the postpartum period. Further studies are needed to clarify this aspect.

Every obstetrician faces challenges related to unexpected events, specific pathophysiology, and appropriate treatment decisions for each individual case. We believe that uterine preservation and fertility are also very important. With early diagnosis and consideration of clinical and hemodynamic conditions, UAE can be an effective and safe option for the treatment of symptomatic UAVM that develops after natural childbirth, even in cases of heavy hemorrhage.

## Data Availability

All data presented here are available from the authors, upon reasonable request.
